# Integrated Automatic Examination Assignment Reduces Radiologist Interruptions: A 2-Year Cohort Study of 232,022 Examinations

**DOI:** 10.1007/s10278-023-00917-7

**Published:** 2024-01-09

**Authors:** Wyanne Law, Admir Terzic, Joshua Chaim, Joseph P. Erinjeri, Hedvig Hricak, Hebert Alberto Vargas, Anton S. Becker

**Affiliations:** 1 Department of Radiology, Memorial Sloan Kettering Cancer Center, New York, NY USA; 2 Department of Radiology, Oncologic Imaging Division, NYU Langone, New York, NY USA

**Keywords:** Radiology workflow optimization, Automatic examination assignment, Turnaround time (TAT), Subspecialization, Health informatics, Systems integration, Workflow efficiency

## Abstract

Radiology departments face challenges in delivering timely and accurate imaging reports, especially in high-volume, subspecialized settings. In this retrospective cohort study at a tertiary cancer center, we assessed the efficacy of an Automatic Assignment System (AAS) in improving radiology workflow efficiency by analyzing 232,022 CT examinations over a 12-month period post-implementation and compared it to a historical control period. The AAS was integrated with the hospital-wide scheduling system and set up to automatically prioritize and distribute unreported CT examinations to available radiologists based on upcoming patient appointments, coupled with an email notification system. Following this AAS implementation, despite a 9% rise in CT volume, coupled with a concurrent 8% increase in the number of available radiologists, the mean daily urgent radiology report requests (URR) significantly decreased by 60% (25 ± 12 to 10 ± 5, t = -17.6, *p* < 0.001), and URR during peak days (95^th^ quantile) was reduced by 52.2% from 46 to 22 requests. Additionally, the mean turnaround time (TAT) for reporting was significantly reduced by 440 min for patients without immediate appointments and by 86 min for those with same-day appointments. Lastly, patient waiting time sampled in one of the outpatient clinics was not negatively affected. These results demonstrate that AAS can substantially decrease workflow interruptions and improve reporting efficiency.

## Introduction

Coordinated patient-doctor visits are an increasingly popular model of healthcare delivery, where all aspects of an outpatient visit, including laboratory tests, imaging studies, and consultations, are conducted on a single day. For example, a patient will undergo imaging in the morning and review these results in the afternoon with their oncologist. This approach offers numerous benefits, including improved patient satisfaction, better health outcomes, and reduced healthcare costs [1]. However, the increased demand for rapid imaging interpretations by radiologists places challenges on radiology departments to devise more efficient workflow management systems.

**Fig. 1:**
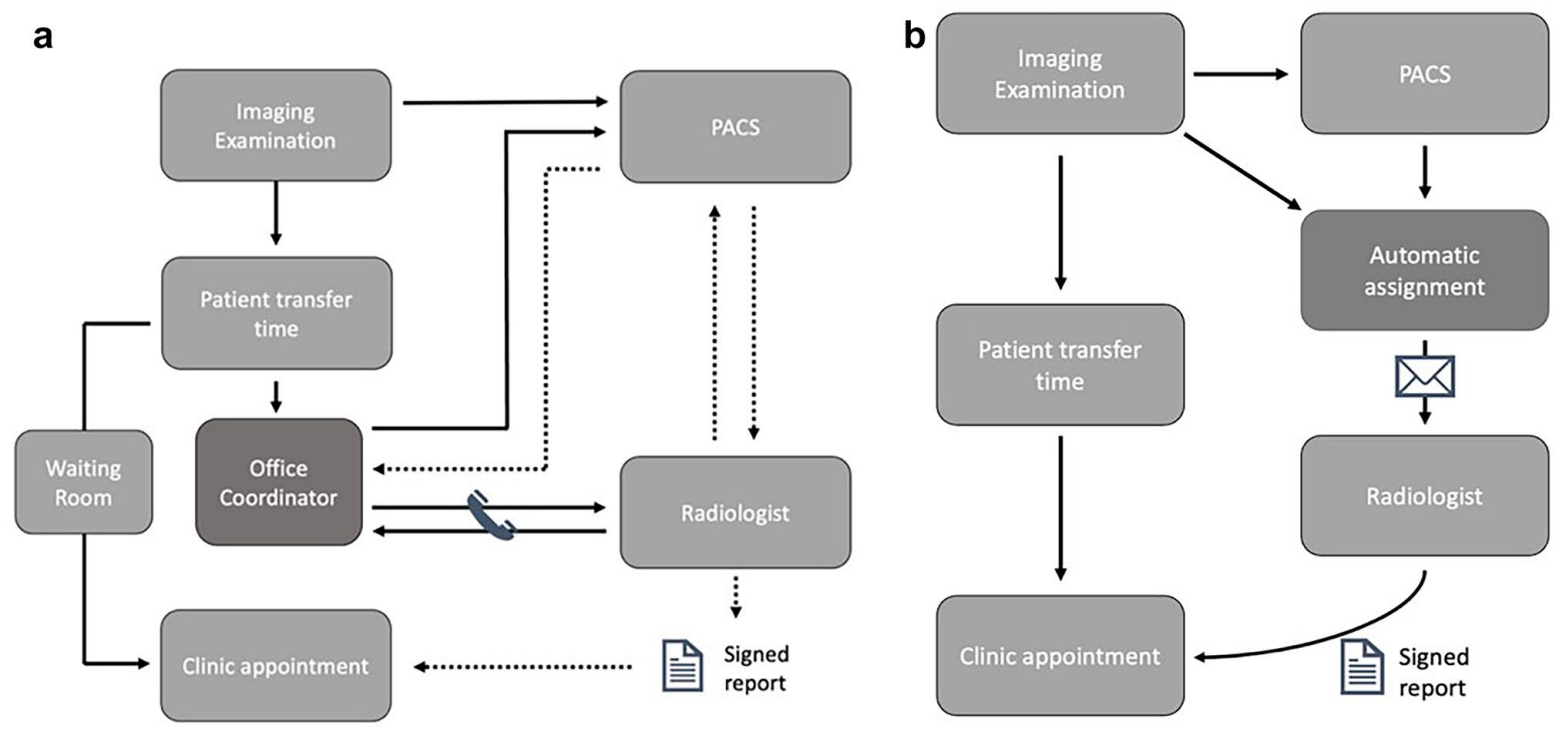
Schematic representation of the workflow for coordinated visits. **a** Legacy workflow involving office coordinators who manually monitor PACS worklists and send requests to the radiology department for read prioritization. **b** New workflow involving integrated automatic assignment and email notifications of upcoming appointments to radiologists

Before integration of our departmental automatic assignment system [1], our radiology service operated in a more “traditional” manner. Examinations were manually picked off a handful of large picture archiving and communications systems (PACS) modality-based worklists by radiologists based on their availability and expertise, requiring radiologists to multitask and often leading to suboptimal prioritization. This manual system, while functional, was also not optimized for the coordinated patient-doctor visit model. As a result, radiologists often faced workflow interruptions from phone calls or emails from office coordinators (Fig. 1a). Such disruptions are known to contribute to burnout [2, 3], diagnostic errors [4], and reduced efficiency [5].

Recognizing these challenges, we integrated a previously developed automated assignment system (AAS), which readily keeps track of unreported examinations and available radiologists [1], with our institutional scheduling software. This integration enabled the AAS to prioritize unreported examinations for patients with upcoming clinic appointments and assign them with priority to available radiologists, aiming to reduce the administrative overhead and workflow interruptions for radiologists (Fig. 1b). Additionally, we launched a microservice notifying radiologists about upcoming appointments of patients in the next 24 h via email whenever they were assigned such examinations.

In the present study, we retrospectively evaluated the effect of the AAS-scheduling integration on the number of urgent read requests (URR) as an indicator for radiologist interruptions. Moreover, we performed an exploratory analysis investigating the effect on patient waiting times for their clinical encounter, as well as the radiology report turnaround times (TAT) during the first 12 months after implementation.

## Methods

### Data Collection and Study Design

This study was Health Insurance Portability and Accountability Act (HIPAA)-compliant and Institutional Review Board-exempt as an institutional quality improvement project. This was a single-center study conducted retrospectively at the body imaging service of a large tertiary cancer center (approx. 90 radiologists) with a highly subspecialized workflow (20 different organ- or disease-specific subspecialized groups, where any given radiologists may be affiliated with more than one group). Data from the 12-month period after workflow intervention from July 1, 2020, to June 30, 2021, was collected (“After”). The 12 months from March 1, 2019, to February 28, 2020, served as a historical control period (“Before”). All types of CTs requested including noncontrast- and contrast-enhanced outpatient examinations performed at our institution. We excluded CTs from the months of March to June 2020 from analysis due to the exceptional and non-representative state of clinical operations during the first COVID-19 wave. The number of CTs and TAT for the corresponding period was extracted from our departmental radiology information system. Daily total number of URR (via phone or email) had been continuously collected by our office coordination team in a shared Microsoft® Excel® spreadsheet.

In the exploratory analysis, patient waiting time (WT), defined as the number of minutes spent in the waiting room after arrival and before entering the gynecologic surgeon’s office, was obtained, which had been recorded by front desk staff in the gynecological surgical outpatient clinic for patients with a recent imaging examination. This clinic was selected due to existing prior collaboration (convenience sample).

### Workflow Intervention

Implementation of the AAS is described in detail in [1]. In short, the AAS queries a database mirroring RIS for unreported examinations. It will then generate an array of medical record numbers (MRNs), which are used to query the hospital scheduling system, thereby receiving all upcoming appointments of patients with unreported examinations. The tables from both queries are joined by MRN and then sorted by appointment time, followed by examination completion timestamp (oldest to newest). Examinations are then distributed starting with the first row. This results in the distribution of examinations with close upcoming appointments first, followed by later appointments and then followed by oldest to newest examination. If there are fewer radiologists than examinations to distribute at a given time point, the most recently completed ones of patients without upcoming appointments would remain undistributed. Additionally, an automatically generated email is sent to the radiologists on the receiving end, so that they know which cases to prioritize.

### Data Analysis

Statistical analysis was performed using R v. 4.0.5. Graphs with a 7-day rolling mean were generated with the {{zoo}} and {{ggplot2}} packages to visually inspect the data. Mean, median, interquartile range, and standard deviation were calculated for descriptive statistics. Two-tailed Student’s *t*-test was performed to compare the mean number of requests, CT examinations, and report turnaround time. Wilcoxon testing was used to compare patient WT before and after automatic assignment. A propensity-matched control cohort was created to compare waiting time differences between patients who had coordinated visits versus patients who did not, using the R package {{matchIt}} v.4.4.0. The factors considered for matching included day of the week, hour of the day, and which gynecologic surgeon they were seeing. A *p*-value of < 0.05 was considered indicative of significant differences.

## Results

**Fig. 2:**
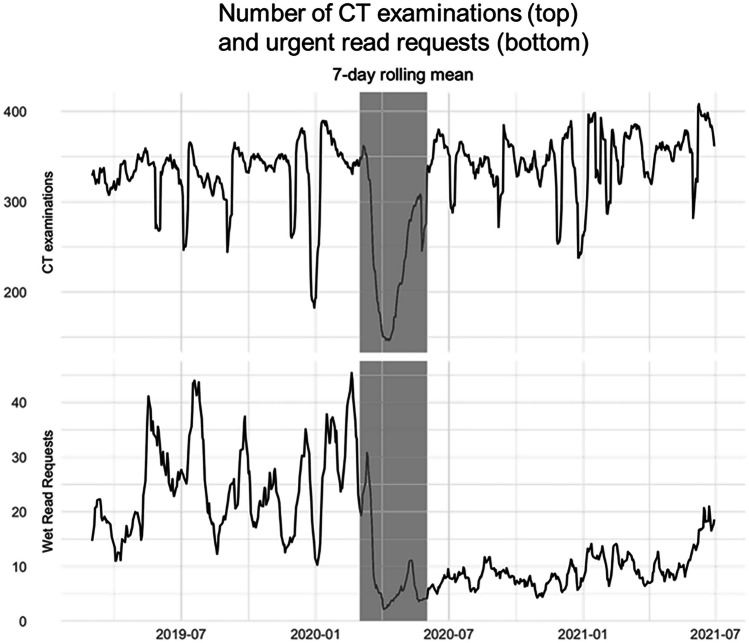
Seven-day rolling mean of CT examinations and urgent read requests for subsequent clinical appointments, before and after implementation of integrated assignment prioritizing reads for patients with upcoming appointments (periods are separated by gray area). The number of urgent read requests significantly decreased (*p* < 0.001), despite increased CT volume (*p* = 0.013)

The total number of CT examinations analyzed was 232,022. The number of CTs increased by 9% from 113,676 to 118,346 in the 12 months after the intervention (*p* = 0.013) and total URR were 6345 (10.6% of total CTs) and 2717 (2.3% of total CTs), respectively, as shown in Fig. 2. The average number of available radiologists per day also increased proportionally by 8% from 27.4 (± 5.0) to 29.6 (± 5.3) (*t* = 4.84, *p* < 0.001), meaning the number of CTs per radiologist was not significantly changed, 17.2 (± 6.6) vs. 16.88 (± 9.7) (*t* =  − 0.42, *p* = 0.67). Despite increased absolute CT volume and virtually unchanged number of CTs per radiologist, the mean number of daily URR significantly decreased by 60.0% (25 ± 12 to 10 ± 5, *t* =  − 17.6, *p* < 0.001). URR during peak days (95^th^ quantile) decreased by 52.2% from 46 to 22 phone calls. These data are summarized in Table 1.

**Table 1:** Comparative analysis of key performance indicators for the two different workflows before and after software integration including number of radiologists per day, number of CTs, and number of phone calls per day

	**Before**		**After**		**Rel. change**	***p*-value**
	Mean ± SD	Median (IQR)	Mean ± SD	Median (IQR)		
**Radiologists/day**	27.4 (± 5.0)	28.0 (23.5–33.0)	29.6 (± 5.3)	30.0 (25.5–35.5)	8%	< 0.001
**CTs/day**	442 (± 77)	455 (394–512)	459 (± 89)	471 (400–554)	9%	< 0.001
**Phone calls/day**	25 (± 12)	22 (12–46)	11 (± 6)	10 (4–22)	− 60%	< 0.001

Median patient WT of 1236 encounters in the gynecological outpatient clinic decreased by 12 (± 48) min (from 30 [IQR 18–51] to 18 [IQR 11–31] min; *p* < 0.001) in the second period, with no significant difference between the two propensity-matched cohorts, as summarized in Table 2.

**Table 2:** Exploratory analysis of median (interquartile range). Patient waiting time in minutes before and after software integration intervention. “Same-day CT” and “no same-day CT” are two propensity score-matched cohorts. The difference between those two cohorts exhibited no significant change (last row)

	**Before**	**After**	***p*-value**
Overall cohort (*n* = 1236)	30 (18–51)	18 (11–31)	< 0.001
Same-day CT (*n* = 618)	33 (22–55)	21 (12–36)	< 0.001
No same-day CT (*n* = 618)	27 (14–43)	18 (9–23)	< 0.001
Difference	6 (− 4–21)	3 (− 6–17)	0.06

Mean TAT significantly decreased by 440 min (from 914 to 474, *p* < 0.001) in patients with no immediate appointment and by 86 min (from 337 to 251, *p* < 0.001) in those with same-day appointment, as summarized in Table 3.

**Table 3:** Mean (± standard deviation) radiology report turnaround times (TAT) showing improvement after the intervention for both groups

**Metric**	**No immediate appointment**, *N* = 126,851			**Same-day appointment**, *N* = 105,171		
	**Before**	**After**	***p*-value**	**Before**	**After**	***p*-value**
Report TAT (minutes)	914 (± 1405)	474 (± 1678)	< 0.001	337 (± 823)	251 (± 555)	< 0.001

## Discussion

Implementation of coordinated AAS fundamentally changes the workflow of the radiologists to achieve the coordinated patient-doctor visit approach while limiting the number of URR. Despite increased CT volumes, we achieved a 60% reduction of URR by integrating the AAS with the hospital-wide scheduling system, accounting for 2.3% of all exams from 10.6%. Report TAT for both patients with and without immediate appointments was decreased by 440 and 86 min respectively. Patient WT was reduced but not significantly changed in our matched cohort.

URR issued by office coordinators or referring physicians in the form of emails and phone calls could cause significant disruption to radiologists’ workflow [2]. Shah et al. quantify the time cost of each interruption at about 6 min [5]. Extrapolating from this approximation from the literature, our intervention may have saved up to 90 min of radiologist time each day.

TAT is often used as a measure of productivity for radiologists. In both groups of patients with same-day appointments and no immediate appointments, TAT significantly decreased after the intervention without changing the individual radiologist’s workload. Rather than waiting for a human office coordinator to sort through studies or the clinicians to alert the radiologist, studies were automatically prioritized as scans were completed.

In our exploratory analysis, we did not find a significant reduction in the patient waiting time at the outpatient oncologic gynecology clinic. We attribute the lack of improvement to factors unrelated to imaging, such as waiting for bloodwork results and, for appointments later in the day, backlog of patients from earlier appointments. This data, however, was unfortunately not available to us for further analysis. Two more caveats need to be noted: First, this sample only represented about a percent of same-day examinations in our study population. Effects in other clinics may have varied. Second and more importantly, our software integration intervention was not aimed at primarily reducing patient waiting time, which may warrant other interventions [6], but at reducing radiology workflow interruptions.

Other institutions have explored different methods of decreasing radiology workflow interruptions. For example, Halsted et al. designed a prioritized worklist based on medical acuity, psychological state, and waiting time, while still allowing radiologists to select freely from the list [7]. Despite increased examination volumes after the intervention, there was no change in TAT, workflow interruptions, or patient satisfaction, hinting at a successful curbing of previously increasing interruptions. Smith et al. used reading room assistants who triaged all phone calls and directed them to the radiologist if appropriate, which contributed to decreased total number of interruptions and increased median time between interruptions [8]. Similarly, Macbeth et al. used an automated phone tree within the emergency radiology reading room that decreased the median number of phone calls and quality improvement of resident workspace satisfaction, feelings of burnout, and burden of interruptions [9]. This highlights that despite the great success of some informatics solutions, a “holistic approach” to workflow optimization remains important.

It is noteworthy that interruptions to the radiological workflow come in many forms, with telephone calls being regarded as one of the most disruptive forms since they funnel disruptions from multiple sources into a single channel [10], and our solution only targeted one specific source. Another limitation of our work is that office coordinators for urgent reads were still required, due to incompleteness of electronically available information. Appointment times may not be available or may change last minute, resulting in cases that were not assigned accordingly. The retrospective nature of the study also limits the evaluation of time spent by office coordinators on this task before and after the intervention. Prospective reproduction at other centers may enable capturing this metric.

For institutions interested in implementing a similar integration of clinical scheduling and radiologic exam distribution, we provide the following guidelines based on our experience:

1. *Needs assessment*: Begin with a thorough assessment of the current workflow, identifying bottlenecks and areas that would benefit most from automation.
2. *Stakeholder engagement*: Engage with radiologists and administrative staff to gather feedback on potential features and concerns.
3. *Technical integration*: Collaborate with IT specialists to ensure the AAS can integrate seamlessly with existing scheduling and reporting software.
4. *Training*: Once implemented, provide comprehensive instructions for all users to familiarize them with the new workflow and the system’s functionalities, for example within existing division or faculty meetings.
5. *Continuous monitoring*: Regularly monitor the system’s performance, gathering feedback for iterative improvements. Consider implementing a feedback loop where radiologists can report any issues or suggest enhancements. One of the shortest feedback loops is to have one or more of the radiologists using the software in the core development team.
6. *Scalability*: Design the system with scalability in mind, allowing for the addition of new features or integration with other systems in the future.

One potential future direction for research in this area is the integration of artificial intelligence (AI) into the automated exam assignment software. Several recent efforts attempt to use artificial intelligence to prioritize examinations based on the image data, for example, suspected clinically significant findings that require urgent management such as intracranial hemorrhage [11]. By integrating this technology into the automated exam assignment software, radiologists could potentially prioritize their work even more efficiently and accurately, leading to further reductions in delays and potentially even better patient outcomes. Another area for future research could be the development of predictive models that can anticipate changes in appointment schedules or patient needs, enabling the software to adjust automatically and ensure that the most urgent cases are always prioritized. Additionally, exploring the use of other forms of automation and digitalization, such as electronic medical record integration or patient self-scheduling, could offer further benefits to the radiology department and hospital as a whole.

## Conclusion

Integration of the departmental automatic assignment software with the institutional scheduling system decreased the number of radiologist interruptions and turnaround time with no negative impact on patient waiting times. Seamless integration of new software systems in the healthcare environment will continue to drive improvements to the radiologic workflow.

## Data Availability

All data contained within article.
